# The Road Traffic Injuries Research Network: a decade of research capacity strengthening in low- and middle-income countries

**DOI:** 10.1186/s12961-016-0084-5

**Published:** 2016-02-27

**Authors:** Adnan A. Hyder, Robyn Norton, Ricardo Pérez-Núñez, Francisco R. Mojarro-Iñiguez, Margie Peden, Olive Kobusingye, Road Traffic Injuries Research Network’s Group

**Affiliations:** Department of International Health & International Injury Research Unit, Johns Hopkins Bloomberg School of Public Health, 615 North Wolfe Street, Suite E-8132, Baltimore, MD 21205 USA; The George Institute for Global Health, Level 13, 321 Kent Street, Sydney, NSW Australia 2000; Secretariado Técnico del Consejo Nacional para la Prevención de Accidentes, Guadalajara 46, tercer piso, Colonia Roma Norte, México Distrito Federal, C.P. 06700 Mexico; Entornos Foundation A.C., Calle 3 #1, Lomas de Atzingo, 62180 Cuernavaca, Morelos Mexico; Unintentional Injury Prevention Unit within the WHO’s Department for the Management of Noncommunicable Diseases, Disability, Violence and Injury Prevention World Health Organization, Avenue Appia 20, 1211 Geneva 27, Switzerland; Makerere University College of Health Sciences, P.O. Box 22864, Kampala, Uganda; Center for Injury Policy and Prevention Research, Hanoi School of Public Health, 138 Giangvo, Badinh, Hanoi, Vietnam

**Keywords:** Capacity development, Global networks, Low- and middle-income countries, Road traffic injuries

## Abstract

Road traffic crashes have been an increasing threat to the wellbeing of road users worldwide; an unacceptably high number of people die or become disabled from them. While high-income countries have successfully implemented effective interventions to help reduce the burden of road traffic injuries (RTIs) in their countries, low- and middle-income countries (LMICs) have not yet achieved similar results. Both scientific research and capacity development have proven to be useful for preventing RTIs in high-income countries. In 1999, a group of leading researchers from different countries decided to join efforts to help promote research on RTIs and develop the capacity of professionals from LMICs. This translated into the creation of the Road Traffic Injuries Research Network (RTIRN) – a partnership of over 1,100 road safety professionals from 114 countries collaborating to facilitate reductions in the burden of RTIs in LMICs by identifying and promoting effective, evidenced-based interventions and supporting research capacity building in road safety research in LMICs. This article presents the work that RTIRN has done over more than a decade, including production of a dozen scientific papers, support of nearly 100 researchers, training of nearly 1,000 people and 35 scholarships granted to researchers from LMICs to attend world conferences, as well as lessons learnt and future challenges to maximize its work.

## Background

Road traffic injuries (RTIs) were the eighth leading cause of death in the world in 2010, whereas in 1990 they occupied the tenth position; this represents an increase of 46% in the total number of deaths due to RTIs during this period [1]. Current trends suggest that, by 2030, RTIs will become the fifth leading cause of death [2]; however, this increase will be disproportionally higher in low- and middle-income countries (LMICs). RTIs not only kill an unacceptable number of people, but millions more suffer non-fatal injuries and a great proportion of them are disabled as a result [2]. As a consequence, since 1990, RTIs have risen from the twelfth leading cause of Disability Adjusted Life Years lost to the tenth leading cause [3]. LMICs also account for the majority of those injuries and disabilities occurring globally.

The Global Forum for Health Research made evident that, of the billions of US dollars spent on health research annually, less than 10% was spent on addressing the health problems relevant to 90% of the world’s population (including RTIs) – the ‘10/90 gap’ [4]. In 2004, in a sentinel report, the World Health Organization (WHO) also highlighted the need for a systematic approach to the prevention and management of RTIs globally [5]. This report summarized relevant cost-effective interventions, and the urgent need for their implementation in LMICs. This report also identified three challenges that have hampered research on implementation of effective road safety interventions and programs in LMICs: insufficient investments in road safety research, inadequate individual and institutional capacity to confront RTIs in a systemic and evidence-based approach, and low participation of professionals from LMICs in key international conferences and meetings.

Scientific research has proven to be useful for preventing RTIs in high-income countries. While there is still a continuing need for research in high-income countries that might also be useful for LMICs, it is important to note that few resources have been allocated for RTI research in many LMICs [6, 7]. As some authors have indicated [7], this is one of the reasons why RTIs were neglected. In response, the UN Road Safety Collaboration was launched in 2004 to promote international collaboration to reduce RTIs. In 2010, the UN General Assembly proclaimed 2011–2020 as the Decade of Action for Road Safety [8]. Over time, this movement has translated into new investments such as those by Bloomberg Philanthropies [9, 10]. In addition, the importance of developing knowledge, skills and capacity for road safety was accepted and considered an important priority [5, 11]. For example, WHO designed and implemented a broad injury prevention program (TEACH-VIP) which is now available as a self-paced, self-administered training resource on the internet, free of charge [12]. WHO also launched an innovative e-mentoring program (MENTOR-VIP) in 2007 [11], which has successfully completed over 50 mentorships globally [12]. However, there are few strong country-based training and capacity development programs.

This paper discusses a global response to these types of challenges by the Road Traffic Injuries Research Network (RTIRN), describing its contribution in developing research capacity and building networking mechanisms for professionals in LMICs. The paper covers the history and founding principles of RTIRN, as well as activities that the network is carrying out with measurable outputs after more than a decade of intensive and international collaborative efforts. The paper concludes with lessons learnt during this time and identifies future challenges in order to maximize the impact of RTIRN.

### What is RTIRN?

RTIRN is an independent partnership of individuals and institutions from around the world that collaborate to advance research on the impact and causes of RTIs in LMICs and to identify appropriate interventions to the problem. The partnership evolved from a June 1999 meeting at the Global Forum for Health Research in Geneva, Switzerland, during which a session on Road Traffic Injuries in Developing Countries stirred significant interest among participants. In April 2000, the first meeting of interested partners was held in Kampala, Uganda, and in 2002 the Network was officially formed. The Global Forum for Health Research was essential in keeping the partners together and provided opportunities to present and discuss their research findings during annual meetings. With the recognition that neglecting RTI research in LMICs significantly contributes to the ‘10/90 gap’ in health research, RTIRN emerged as a distinct effort to address this disequilibrium.

The vision of RTIRN is to reduce the burden of RTIs, particularly in LMICs, through conducting, promoting and utilizing research. Its goal is to establish networking mechanisms and assist in the creation of partnerships between RTI researchers and institutions globally to support research and research capacity in LMICs. In order to achieve this, RTIRN has set a series of specific objectives and basic principles of operation (Table 1). The objectives focus on advocating, conducting and producing research in and for LMICs. The principles of operation reflect the intent of the founders to create a network which is effective, open and sensitive to the problem and people it engages.

**Table 1:** Objectives and principles of Road Traffic Injuries Research Network

Specific objectives
• To advocate for research to reduce the burden of road traffic injuries (RTIs) in LMICs
• To set priorities for RTI research in LMICs
• To help develop capacity for RTI research in LMICs
• To promote investments for RTI research in LMICs
• To facilitate communication between partners involved in RTI research in LMICs
• To conduct strategic research on RTIs in LMICs
• To disseminate and promote the application and utilization of research to reduce the burden of RTIs in LMICs
Basic principles of operation
• Transparency in its creation and governance
• Effective governance, accountable to its Partners
• Sensitivity to gender, language, diversity of disciplines, differential needs, and policies of related sectors, and to their implications for its mode of work
• Complementing rather than duplicating existing activities, and in particular focusing energies where an international entity has a comparative advantage
• Ensuring special attention to equity concerns and the capacity building needs of LMICs

**Table 2:** Distribution of Road Traffic Injuries Research Network (RTIRN) programs by regional and topical characteristics

		Small Grant Program	Junior Researcher Program	Senior Researcher Program	Regional Workshops	Webinars
WHO Region	Africa	7	2		2	
	Americas		3	2	3	
	Eastern Mediterranean	1	1	1	1	
	South-East Asia	5			1	
	Western Pacific		3	1	3	
	Europe					
	All or NA					5
Topic	Epidemiological/methods oriented	7	6	4	3	2
	Health systems/program oriented	6	3		7	3
Risk factors^a^	Alcohol	1	3			
	Speeding	2	1			
	Seatbelt/child restraint use		1			
	Helmet use				1	
	Distraction/inattention		1	1		
	Road design/structure	3	3			
	Visibility	1				
	All or NA	8	3	3	9	5
Five Pillars of the UN Decade of Action	1. Road Safety Management	1			1	2
	2. Safer Roads and Mobility	3	3			
	3. Safer Vehicles					
	4. Safe Road Users	3	4	1	1	
	5. Post-crash Response		1		1	
	All or NA	6	1	3	7	3
Road user^a^	Pedestrian	1	1		1	
	Cyclists		1			
	Motorcycle user	2	1		3	
	Car occupant		3	1		
	Public transport					
	Commercial transport/drivers	1	1			
	All or NA	9	2	3	6	5
Sex of recipient/beneficiaries	Male	9	6	3		
	Female	4	3	1		
	Both				1236	200
	Total activities	13	9	4	16	5

^a^Studies could be classified into more than one category

**Fig. 1:**
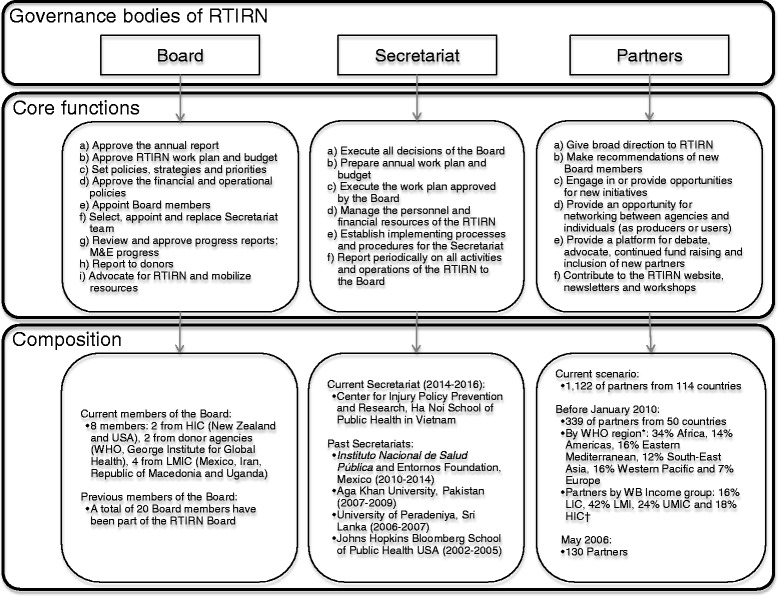
Structure of the Road Traffic Injuries Research Network. RTIRN, Road Traffic Injuries Research Network; HIC, High-income countries; WHO, World Health Organization; LMICs, Low- and middle-income countries; LIC, Low-income countries; LMI, Lower middle-income countries; UPMIC, Upper middle-income countries. Notes: * This corresponds to WHO’s definition of region groupings, available at: http://www.who.int/healthinfo/global_burden_disease/definition_regions/en/ † World Bank’s Classification of Countries’ income of February 2014, available at: http://data.worldbank.org/about/country-classifications/country-and-lending-groups

RTIRN is comprised of (1) the Board; (2) the Secretariat; and (3) RTIRN partners, which together make up its constituent bodies (Fig. 1). The RTIRN Board is composed of 10–15 members; usually two high-income country health researchers, four LMIC health researchers, three representatives of funding agencies and a non-health sector researcher. The Board represents the highest policy- and decision-making body of RTIRN. Amongst its core functions are approvals of agreements with donors, the development of work plans and overseeing the activities of the Secretariat. A key activity is to help mobilize resources in order to implement different programs of the Network. Currently, RTIRN has eight Board members; a total of 20 leaders from across the world have been Board members at some point since 2002.

The RTIRN Secretariat is hosted by a national or international organization selected by the Board for a 2-year term. The function of the Secretariat is to prepare work plans and to execute all activities proposed and decisions made by the Board. The Secretary manages personnel and financial resources, organizes meetings and reports on a regular basis to the Board. To date, five different teams of Secretariats (based in Sri Lanka, Pakistan, Mexico and Vietnam) have worked for the Network, contributing to achieving its mission and objectives.

**Fig. 2:**
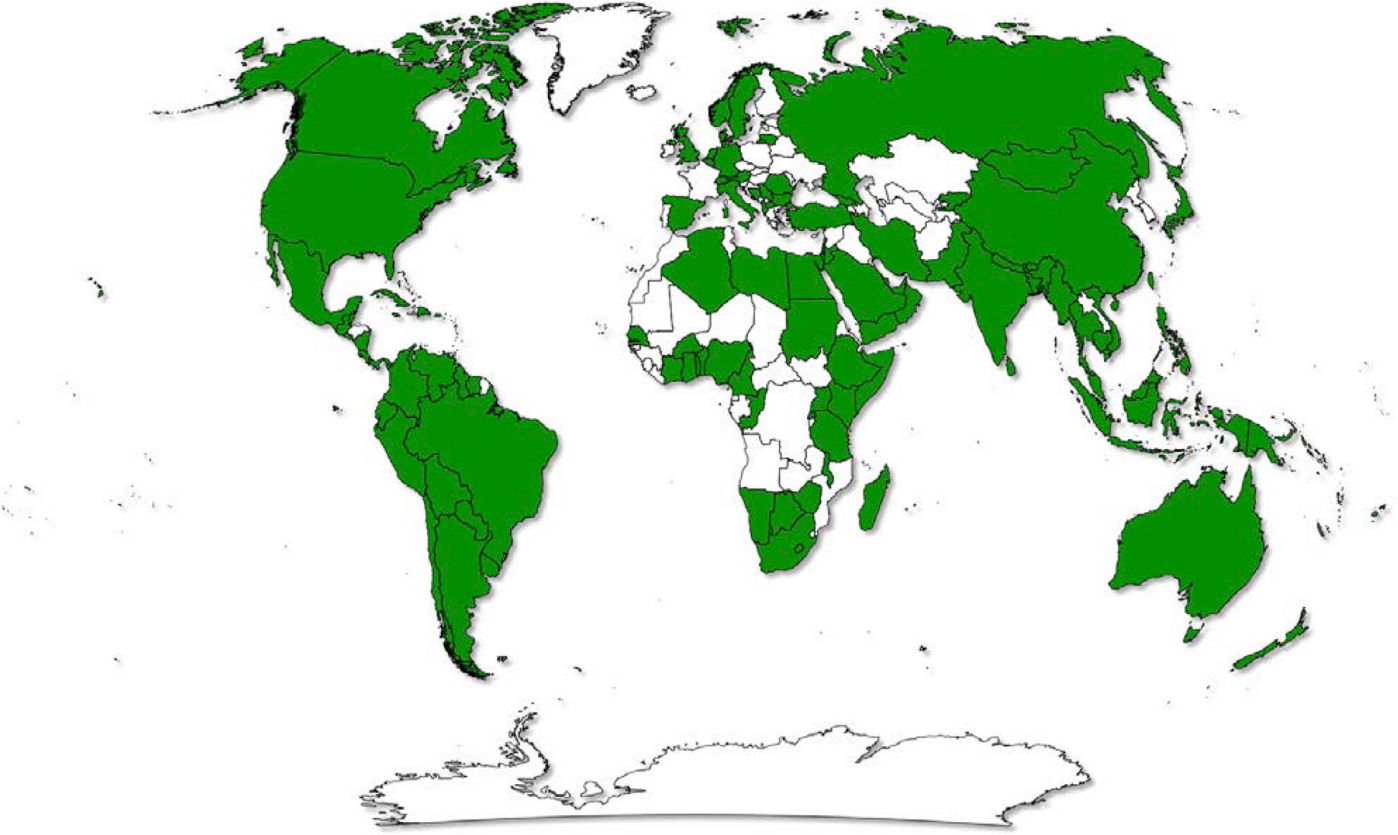
Global distribution of Road Traffic Injuries Research Network (RTIRN) partners (Green denotes countries with at least one RTIRN partner)

**Fig. 3:**
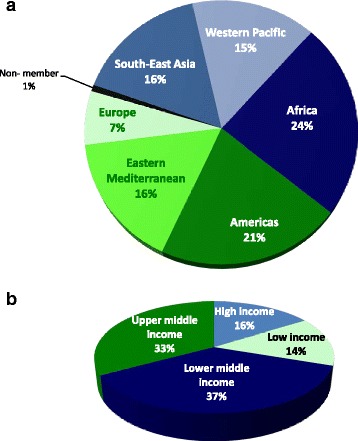
Distribution of current Road Traffic Injuries Research Network partners, March 5, 2015 (n = 1,122) (**a**) By World Health Organization Regions: (**b**) By World’s Bank 2014 Income Classification

Finally, the RTIRN partnership is a collection of individuals collaborating on RTI research in LMICs. Partners represent independent academics, individual researchers, research institutions, students, users of research, and both public and private agencies. Partners play an important role in providing broad direction to RTIRN. They contribute in various ways such as sharing information and opportunities with the RTIRN community and engaging in different projects and activities of the Network. As of March 5, 2015, there were 1,122 RTIRN partners, representing 114 countries (Fig. 2). Amongst them, 13.9% are from low-income countries, 37.0% from low-middle income countries, 32.9% from upper-middle income countries, and 16.2% from high-income countries. The WHO region with the most partners is Africa (24.1% of partners), followed by the Americas (21.2%, Fig. 3). Of note, partnership is cost-free.

### Strategies to build RTI research capacity and networking

One of the key activities of the Network has been to support professionals and researchers in LMICs generate evidence and help build national capacity. RTIRN has supported two rounds of a small grants program, several research projects and a multi-country initiative (Table 2).

#### Small grants program

RTIRN has been able to support two rounds of a small grants program, which was an open call for researchers in LMICs to apply for any road safety-related topic and secure a peer-reviewed award. RTIRN awarded 13 grants, which allowed 56 professionals to conduct research on priority topics such as road design, alcohol use, inappropriate speed and vulnerable road users, e.g. pedestrians and motorcyclists (Table 2). The demand for such grants is high; for example, the 2013 call received a total of 113 proposals from 45 different LMICs. This highlights both the reach of the RTIRN partner base and the need to invest resources for research on RTIs.

#### Junior and senior researchers programs

A strategy used by RTIRN to support research capacity development in LMICs has been to support career development of professionals in road safety. RTIRN awarded 1-year grants to early career (junior) researchers and postgraduate students from LMICs working in the field of injury prevention and road safety in their country of origin (Table 2). These junior professionals used these grants to undertake research projects relevant to RTIs in Africa, Americas, Eastern Mediterranean and Western Pacific regions, and were supervised by local experts. These projects provided new insights regarding context-specific risk factors from alcohol to infrastructure and further measurement of the burden of RTIs, especially on vulnerable road users. A compiled report of the main findings of each study was published by RTIRN and eight were also published in scientific peer-reviewed journals [13–20].

RTIRN also offered grants to senior researchers from LMICs to support a sabbatical to provide secure time to write papers and proposals, or visit and develop collaborations with other international colleagues [21]. The program helped senior researchers expand their knowledge and experience by sharing ideas and meeting other colleagues and renowned researchers, as well as interacting with young professionals and students in the field of RTI prevention. For example, one researcher worked on a burden of disease study in Argentina while another spent time in WHO’s Department of Violence and Injury Prevention and Disability, collaborating on the Eastern Mediterranean regional status report on road safety (Table 2) [3, 22]. Four scientific papers resulted from these grants [19, 20, 23, 24].

#### Multi-country study on non-standard helmets

The use of non-standard motorcycle helmets has the potential to undermine multinational efforts aimed at reducing the burden of RTIs associated with motorcycle crashes. However, little was known about the prevalence or factors associated with their use; therefore, RTIRN launched a multi-country study in collaboration with nine institutions in LMICs from five different WHO regions. The study conducted cross-sectional surveys, market surveys, and reviewed legislation and enforcement practices around non-standard helmets, generating evidence to inform key stakeholders on this important issue. The implementation model for this study allowed local researchers and practitioners to gain experience in RTI research together with international experts. This model allowed for optimal learning for researchers through inbuilt mentoring and led to a final publication in the scientific literature [25].

#### Regional workshops and scientific meetings

RTIRN organizes regional workshops designed to train local and national participants from different backgrounds working on road safety, including those from government, NGOs, and academia. Led by a team of international road safety experts with broad experience in road safety research and training, these workshops were organized to cover several components of road safety (risk factors to enforcement) to ensure that targeted regions will benefit from the latest information [8]. Participants also have the opportunity to network and make important connections to support their current and future research. Thus far, RTIRN has conducted 16 workshops in countries like Sri Lanka, Malaysia, Pakistan, Kenya, Ghana, Mexico, Indonesia, New Zealand and Brazil (Table 2). Approximately 1,236 people attended these regional workshops and provided positive feedback; for example, in a workshop of 36 participants, 34 rated the workshop as excellent or outstanding.

The Network also encouraged networking by providing scholarships to attend the World Conferences on Injury Prevention and Safety Promotion. This included 13 young professionals from LMICs supported to the seventh world conference in Vienna in 2004 and 22 to the tenth conference in London. In fact, for 2010, this represented 30% of all scholarships provided during the world conference. These 35 scholarships represented an average investment of only US$ 1,850 per person.

#### Webinars

In 2010, RTIRN began a webinar series that brings state-of-the-art lectures and speakers to LMIC researchers at a very low cost (Table 2). To date, with the support of institutions like WHO, the National Institute of Public Health in Mexico, The George Institute for Global Health, the University of Auckland, Youth for Road Safety, and the Johns Hopkins International Injury Research Unit, RTIRN has organized five webinars with over 200 attendees. Topics covered included disabilities, capacity building for injury prevention, and road safety legislation. These webinars are available on RTIRN’s website free of charge for future reference for any road safety professional; 132 additional participants have downloaded these webinars already.

#### Newsletters

The RTIRN newsletter is one way for the Secretariat and the Board to be held accountable for the activities conducted on behalf of RTIRN. Partners are informed on a regular basis of the main events and opportunities available through RTIRN. The newsletter also provides a platform through which both young and established researchers from all over the world can share experiences on road safety research. Since 2004, RTIRN has published 199 contributions in 33 newsletters. In order to make this information available to the widest audience, RTIRN translated the newsletters to Spanish, French, Farsi (Persian), Vietnamese, Chinese, and Portuguese. All the newsletters are available on the RTIRN webpage, as well as through listserv distribution. Since April 2011 (when monitoring of downloads began), of the 59 different versions of the newsletter, a total of 1,867 have been downloaded. In 2011, RTIRN also started an annual Best Contribution Award to stimulate partners to keep sharing their work with the Network; this award has been won by two individuals.

#### Utilizing social media

RTIRN developed a website (www.rtirn.net) to introduce the Network to a wider community and promote accountability to partners and funders by displaying activities being carried out. The site serves as an information centre for those interested in road safety, including upcoming events and publications produced by the Network and its partners. The website also showcases plans and actions in favour of the UN Decade of Action for Road Safety 2011–2020. The Online Forums on the website allows partners to discuss and comment on topics of common interest.

In March 2011, RTIRN also launched Facebook (www.facebook.com/RTIRN) and Twitter (@RTIRN) accounts. All of these provide a venue for the exchange of ideas and information, and disseminate products and results of activities. The Network’s Facebook page has nearly 422 fans, receives an average of 435 visits per year and there are around three new posts/shares per week; while the RTIRN Twitter account has more than 381 followers and we follow 151 partners.

#### RTIRN listserv

The RTIRN listserv is used to share different opportunities from grant applications, conferences and webinars to job announcements; over time, many partners have indicated that the listserv is key to helping them expand their reach to other researchers and road safety professionals. This allows RTIRN to facilitate networking and interaction amongst partners with different expertise from different contexts.

## Conclusions

The work carried out by RTIRN has not gone unnoticed at a global level. In 2004, WHO acknowledged the important work of RTIRN in capacity building [5]; RTIRN was included in three of the UN Secretary General’s reports on improving road safety [26–28]; and in April 2011, RTIRN received The Prince Michael International Road Safety Award in recognition of its outstanding contribution to the improvement of road safety internationally.

RTIRN has continued to grow steadily and more partners have joined each year – an indication that partners both believe in the work carried out by RTIRN but also recognize that there are benefits in being part of the network. As mentioned above, at least 12 scientific papers have been published [6, 13–20, 23–25] due to work supported by the network and approximately 100 researchers and students have been supported to conduct relevant research. A total of 1,236 people have attended regional workshops or scientific meetings, about 300 people have participated in webinars, and 35 professionals were funded to attend the international conferences (Table 2). In addition, donor agencies have continued to fund RTIRN, and it has raised more than US$ 1 million to conduct its activities as a leading network promoting research and capacity development in LMICs. It has become the ‘go to’ institution for students and professionals to obtain research information and support for RTIs in LMICs.

RTIRN appears to be the only academic- or research-oriented, free, global network for those working on road safety, and thus it has closed an important gap, especially for LMICs. The Network is comprised of a large group of committed road safety professionals who have been able to support this international effort for more than a decade. Since its inception, RTIRN has promoted the conduct and implementation of research for reducing the impact of RTIs in LMICs. RTIRN recognizes that one of the main concerns in road safety research is the lack of support for research and has devoted itself to securing funds to support researchers and their institutions. This work depends on the voluntary time and commitment of Board members, Secretariat staff, and partners. Thus, it is important that the work of RTIRN be widely disseminated so that funding agencies, donors and philanthropic institutions will invest their resources with the Network, which will help ensure continued success in the future.

RTIRN has faced several challenges, and continues to do so, with creativity. First, the core team is voluntary and the commitment of board members can be potentially limited by other competing responsibilities. However, the limitation of board terms, commitment of individuals and strong interest by senior professionals has been sustained over 12 years. Second, it is important to mentor the next generation of board members and one approach being explored is the integration of young professionals into the Board. This would create incentives for young professionals to work hard and learn from others in the field, thus helping build the capacity. Third, like other networks, RTIRN needs to keep partners interested and engaged in activities. A recent innovation has been the move from online web-based forums to enhanced presence on social media such as Facebook and Twitter. Finally, encouraging speakers to share their experience through webinars has proved challenging at times – many senior professionals do not feel comfortable working with this type of technology, which does not allow for direct interaction with the audience. However, recruitment of experienced professionals used to online teaching has helped launch several webinars.

In order to achieve the UN Decade of Action for Road Safety 2011–2020 objectives, more efforts should be made in terms of capacity building and allocating resources for RTI research in many LMICs. Both scientific research and capacity development have proven to be useful for preventing RTIs in high-income countries and, of course, research continues to play an important role in those regions. RTIRN, as the only academic- or research-oriented, free, global network for those working on road safety, filled the gap by creating a dedicated group for road safety researchers, and has motivated funding agencies to invest key resources in support of road safety professionals from LMICs [7]. As suggested by WHO, LMIC governments can take advantage of RTIRN’s ability to generate scientific evidence and to build a new generation of road safety professionals at country level [2, 5, 29]. The extent to which RTIRN’s work has contributed to positive change in LMICs is difficult to evaluate but the continued growth in partners after a dozen years is a sign that the need for capacity development for RTI research remains relevant today.
